# Pulsed electromagnetic field prevents lumbar bone loss in orchiectomy mice without altering systemic iron metabolism

**DOI:** 10.3389/fbioe.2025.1684162

**Published:** 2025-11-07

**Authors:** Jiancheng Yang, Yan Feng, Mingming Pan, Yuhong Zeng

**Affiliations:** Department of Osteoporosis, Honghui Hospital, Xi’an Jiaotong University, Xi’an, China

**Keywords:** pulsed electromagnetic field, male osteoporosis, orchiectomy, iron metabolism, bone loss

## Abstract

**Background:**

The therapeutic efficacies of pulsed electromagnetic fields (PEMFs) for postmenopausal osteoporosis are well established; however, their effects on male osteoporosis remain uncertain. Osteoporosis development is often associated with dysregulation of systemic iron metabolism. This study aimed to investigate the therapeutic effects of PEMFs on orchiectomy (ORX)-induced osteoporosis in mice and determine whether the mechanism involves regulation of systemic iron metabolism.

**Methods:**

Twenty-four 10-week-old male C57BL/6J mice were divided into the sham, ORX, and ORX + PEMF groups (n = 8 each). The ORX + PEMF group received PEMF treatment (15 Hz, peak of 3.82 mT, 40 min/d for 2 months) starting 2 weeks post-surgery. The bone loss was assessed via micro-computed tomography, biomechanical testing, dynamic histomorphometry, and TRAP staining. The systemic iron metabolism was evaluated via Prussian blue staining, atomic absorption spectrometry, and serum ELISA (hepcidin, ferritin).

**Results:**

PEMF treatment partially prevented ORX-induced bone loss in the lumbar spine, as evidenced by significant improvements in the microstructure and mechanical properties of the bone, along with partially restored bone formation and suppressed bone resorption compared to the untreated ORX mice. However, PEMF application showed no significant protective effects on the femoral bone microstructure or cortical parameters. Crucially, PEMF treatment did not alter the markers of systemic iron metabolism, meaning that no changes were observed in the tissue iron deposition, iron content, serum ferritin level, serum hepcidin level, or liver hepcidin expression.

**Conclusion:**

PEMF therapy exerts site-specific protective effects against ORX-induced osteoporosis in the lumbar spine of male mice to partially restore bone formation and reduce resorption. This therapeutic efficacy is independent of any alterations in the systemic iron metabolism. The findings of this study support PEMF application as a potential physical therapy for male osteoporosis while highlighting the need for site-optimized strategies.

## 1 Introduction

Osteoporosis is a disease with a high global prevalence. According to statistics, the global prevalence of osteoporosis is 19.7%, with women being affected at a significantly higher rate (27.4%) than men (10.6%) (Xiao et al., 2022). Consequently, osteoporosis has been historically regarded as a female-centric disease, with all research, intervention trials, and clinical guidelines predominantly focusing on women (Fuggle et al., 2024; Vilaca et al., 2022). However, the prevalence of osteopenia is slightly higher in men (44.8%) than women (42.1%) (Naso et al., 2025). If left untreated, osteoporosis can ultimately lead to osteoporotic fractures. Although the prevalence of osteoporosis in women is nearly thrice that in men, the occurrence of fractures in men aged 40 years and above is comparable to that in women (Wang et al., 2021a). In many populations, it has been estimated that one in five men over the age of 50 may experience an osteoporotic fracture during their remaining lifetime (Kanis et al., 2000; Byberg et al., 2011). Therefore, osteoporosis treatment in men should not be overlooked.

In recent years, there have been extensive research efforts into the application of magnetic fields in orthopedics, as there are proven significant therapeutic effects on orthopedic diseases, such as osteoporosis (Xu et al., 2025; Liu et al., 2025; Yang et al., 2023). Based on their principles of occurrence and state, magnetic fields can be divided into two main forms as static and dynamic. The most common form of dynamic magnetic field is the pulsed electromagnetic field (PEMF), which has been widely used as a clinical adjunctive treatment against primary osteoporosis (Zhu et al., 2022). However, extant studies have focused mostly on the treatment of postmenopausal osteoporosis with PEMFs, while very few studies have explored the therapeutic effects against male osteoporosis.

Iron is an essential element in the human body that is involved in many important physiological processes. However, iron is also a reactive metal that catalyzes the production of excess reactive oxygen species (ROS) and causes cellular damage, leading to various diseases (Roemhild et al., 2021). A large body of clinical evidence and animal model studies have confirmed that iron overload or accumulation is closely related to osteoporosis (Ru et al., 2023; von Brackel and Oheim, 2024). Our previous studies showed that applying a magnetic field of a certain intensity can affect the activities and functions of osteoblasts, osteoclasts, and osteocytes by regulating cellular iron metabolism (Yang et al., 2018; Dong et al., 2019; Yang et al., 2021a). Therefore, magnetic fields have regulatory effects on cellular and systemic iron metabolism, which are possible mechanisms by which magnetic fields act on bone tissue (Zhen et al., 2025; Zhen et al., 2024; Zhang et al., 2023). In this study, we investigated the therapeutic effects of PEMFs on male osteoporosis and the regulation of systemic iron metabolism in elderly male osteoporosis patients as well as the orchiectomy (ORX)-induced male mouse osteoporosis model; the aim of this work was to clarify whether PEMFs can be used to treat male osteoporosis by regulating iron metabolism.

## 2 Methods

### 2.1 Animal grouping and treatment

Twenty-four 10-week-old male C57BL/6J mice were housed at an ambient temperature of 25 °C under 12-h artificial light/dark cycles with free access to food and water. All mice were randomly assigned to one of three groups (n = 8 per group) as follows: sham group (removal of peritesticular fat pads); ORX group (bilateral orchiectomy); ORX with PEMF treatment (ORX + PEMF) group (bilateral orchiectomy followed by PEMF treatment). After a 2-week recovery period following surgery, the mice in the ORX + PEMF group received treatment with the XT-2000B PEMF stimulator (Aerospace Optoelectronic Technology Development (Tianjin) Co., Ltd., Tianjin, China). This apparatus incorporates a large set of Helmholtz coils, and the entire mouse cage is positioned at the center of the set of coils to apply a uniform magnetic field. Thus, the magnetic field intensity and frequency experienced by the mice remain consistent throughout their bodies. Briefly, each pulse lasted 0.2 ms and was repeated at a frequency of 15 Hz. Within the treatment area, the magnetic field was delivered vertically. The flux density of a single burst started at a peak of 3.82 mT and declined to 0 mT over 0.2 ms. The ORX mice in the PEMF group underwent treatment continuously for 40 min per day over three treatment courses lasting 10 d each. The treatment frequency per course was as follows: once daily for course 1, alternate days for course 2, and once every 2 d for course 3. The total duration of the PEMF treatment was 2 months. All animal experimental procedures were approved by the Experimental Animal Ethics and Welfare Committee of Honghui Hospital, Xi’an Jiaotong University (no. 202212014).

### 2.2 Micro-computed tomography (micro-CT) scanning

The mouse femora and lumbar vertebrae (L3) were scanned using a high-resolution micro-CT scanner (SkyScan 1176, Bruker, Karlsruhe, Germany), and the acquired scans were reconstructed into three-dimensional (3D) images using NRecon software (Bruker). The trabecular bone parameters (bone volume fraction, BV/TV; trabecular thickness, Tb.Th; trabecular number, Tb.N; trabecular separation, Tb.Sp; connectivity density, Conn.D; structure model index, SMI) of the femur and L3 as well as the cortical bone parameters of the femur (cortical thickness, Ct.Th; cortical porosity, Ct.Po; cortical area, Ct.Ar; cortical area fraction, Ct.Ar/Tt.Ar; polar moment of inertia, PMI) were analyzed using CTAn software (Bruker). Additionally, the bone mineral densities (BMDs) of the cancellous bones in the femur and L3 as well as the tissue mineral density (TMD) of the femoral cortical bone were determined using CTAn.

### 2.3 Bone biomechanical testing

The frozen tibiae were thawed to room temperature before removing the soft tissues. Then, the structural mechanical properties of the tibiae, including ultimate load, stiffness, and bending energy absorption, were assessed via three-point bending tests using a material mechanics testing equipment (Dong Ri, Guangdong, China). Subsequently, the outer diameter, inner diameter, wall thickness, and marrow cavity size at the fracture site were measured under a stereomicroscope (NOVEL, Nanjing, China). The material mechanical parameters, including ultimate stress, elastic modulus, and toughness, were calculated using previously described methods (Yang et al., 2021b). Next, the lumbar vertebrae (L4) were harvested, soft tissues were removed, and mechanical properties were tested via axial compression. The loading platen was placed perpendicular to the vertebral cross-section and compressed downward at a constant speed of 1 mm/min. The mechanical parameters of the vertebral body, including ultimate load, stiffness, and elastic modulus, were calculated.

### 2.4 Histochemical staining

The lumbar vertebrae L3 analyzed using micro-CT were subsequently decalcified, embedded in paraffin, and sectioned. The bone sections were stained for tartrate-resistant acid phosphatase (TRAP; ServiceBio, Wuhan, China) to assess osteoclast activities on the cancellous bone surfaces. The number of osteoclasts per bone surface (N.Oc/BS) and osteoclast surface per bone surface (Oc.S/BS) were calculated.

### 2.5 Dynamic bone histomorphometry

Calcein (35 mg/kg bodyweight; ServiceBio) was injected intraperitoneally 10 d and 3 d prior to euthanasia. The femora were then fixed, dehydrated, embedded in methyl methacrylate, and sectioned (50 µm thickness). The distance between the two fluorescent labels was measured using ImageJ software (National Institutes of Health, Bethesda, MD, United States). The mineral apposition rate (MAR) and bone formation rate per bone surface (BFR/BS) were calculated.

### 2.6 Prussian blue staining

Iron depositions in the liver, spleen, and lumbar vertebra L3 were evaluated via 3,3′-diaminobenzidine (DAB)-enhanced Prussian blue staining. Here, DAB is oxidized by hydrogen peroxide decomposed from the Prussian blue precipitate and undergoes successive oxidative polymerization as well as cyclization to form a visible brown precipitate. This DAB enhancement significantly amplifies the iron staining signal, facilitating observation and analysis.

### 2.7 Iron content testing

The iron content of the tissues was tested according to a method reported previously (Guo et al., 2024). Briefly, the liver, spleen, and lumbar vertebra L5 were dehydrated in an oven at 180 °C for 6 h, and their dry weights were measured using an electronic balance. The dried samples were then incinerated in a muffle furnace (Taisite, Tianjin, China) at 600 °C to obtain ash. Subsequently, concentrated nitric acid (65%) was added to the ash sample and incubated on a constant-temperature heating plate at 70 °C for 2 h to ensure complete digestion. The digested samples were diluted using a 0.1% solution of potassium chloride (KCl), and the iron levels were detected by atomic absorption spectrometry (AAS; Analytik Jena, Germany) and normalized to the tissue dry weight.

### 2.8 Serum biochemical assay in mice

The serum levels of CTX, P1NP, ferritin, and hepcidin in the mice were measured using commercially available mouse ELISA kits (Elabscience, Wuhan, China) according to manufacturer instructions.

### 2.9 Immunofluorescence staining

The paraffin sections of the liver tissue were dewaxed and immersed in a citrate antigen retrieval solution (pH 6.0) for antigen recovery. The sections were then incubated in 3% hydrogen peroxide solution in the dark to block endogenous peroxidase activity. Subsequently, the sections were blocked with 3% bovine serum albumin (BSA) at room temperature for 30 min. The slides were next incubated with a primary antibody against hepcidin (DF6429, Affinity, OH, United States) overnight at 4 °C. After washing with phosphate-buffered saline (PBS), the sections were incubated with a corresponding species-specific FITC-conjugated secondary antibody at room temperature for 50 min. The nuclei were counterstained with 4′,6-diamidino-2-phenylindole (DAPI; ServiceBio). After dehydration, the sections were mounted using an anti-fade mounting medium and visualized under a fluorescence microscope. The fluorescence intensity was quantified using ImageJ software.

### 2.10 Statistical analysis

All experimental findings are presented as mean ± standard deviation (SD). We used GraphPad Prism (GraphPad Software, San Diego, CA, United States) for the statistical analysis. Differences among multiple groups were analyzed using one-way ANOVA followed by a post-hoc multiple comparison test. A *p*-value of less than 0.05 was considered to be statistically significant.

## 3 Results

### 3.1 PEMF application did not prevent ORX-induced femoral bone loss in male mice

**FIGURE 1:**
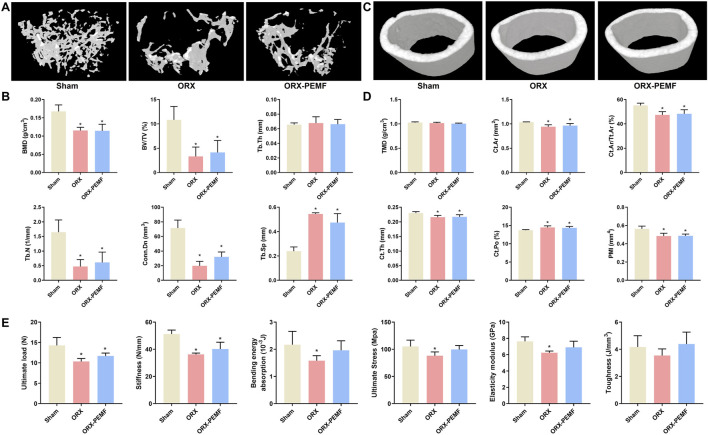
Effects of pulsed electromagnetic field (PEMF) application on femoral microstructure and tibial biomechanics in orchiectomy (ORX) mice. **(A)** Three-dimensional images of trabecular bone. **(B)** Bone mineral density (BMD) and structural parameters of trabecular bone, including bone volume fraction (BV/TV), trabecular thickness (Tb.Th), trabecular number (Tb.N), connectivity density (Conn.D), and trabecular separation (Tb.Sp). **(C)** Three-dimensional images of cortical bone. **(D)** Tissue mineral density (TMD) and structural parameters of cortical bone, including cortical area (Ct.Ar), cortical area fraction (Ct.Ar/Tt.Ar), cortical thickness (Ct.Th), cortical porosity (Ct.Po), and polar moment of inertia (PMI). **(E)** Biomechanical properties of the tibia, including ultimate load, stiffness, bending energy absorption, ultimate stress, elastic modulus, and toughness, were examined via three-point bending tests. The data are shown as mean ± SD (n = 8). *p < 0.05 vs. sham.

The micro-CT analysis revealed that the ORX procedure significantly reduced the BMD of femoral cancellous bone and impaired the trabecular microstructure in male mice, as evidenced by the significant decreases in BV/TV, Tb.N, and Conn.Dn along with significant increase in Tb.Sp compared to the sham group (Figures 1A,B). However, PEMF treatment showed no significant effects on the BMD of femoral cancellous bone or trabecular microstructure in ORX mice. Similarly, the ORX procedure resulted in a significant decrease in the TMD of femoral cortical bone and impaired the cortical microstructure, as characterized by the significant decreases in Ct.Ar, Ct.Ar/Tt.Ar, Ct.Th, and PMI along with a significant increase in Ct.Po (Figures 1C,D). Moreover, PEMF treatment had no significant effects on the femoral cortical TMD and cortical microstructure parameters in ORX mice.

The three-point bending tests revealed reduced biomechanical properties of the tibia of ORX mice. In terms of the structural mechanical properties, the ORX procedure significantly decreased the ultimate load, stiffness, and bending energy absorption. In this case, PEMF intervention did not prevent the reduction in ultimate load or stiffness but mitigated the reduction in bending energy absorption (Figure 1E). With regard to the material mechanical properties, the ORX procedure significantly decreased the ultimate stress and elastic modulus, which were prevented by PEMF intervention. These data indicate that PEMF application partially prevented the impairment of tibial biomechanical properties induced by the ORX procedure.

### 3.2 PEMF application partially prevented ORX-induced lumbar vertebral bone loss in mice

**FIGURE 2:**
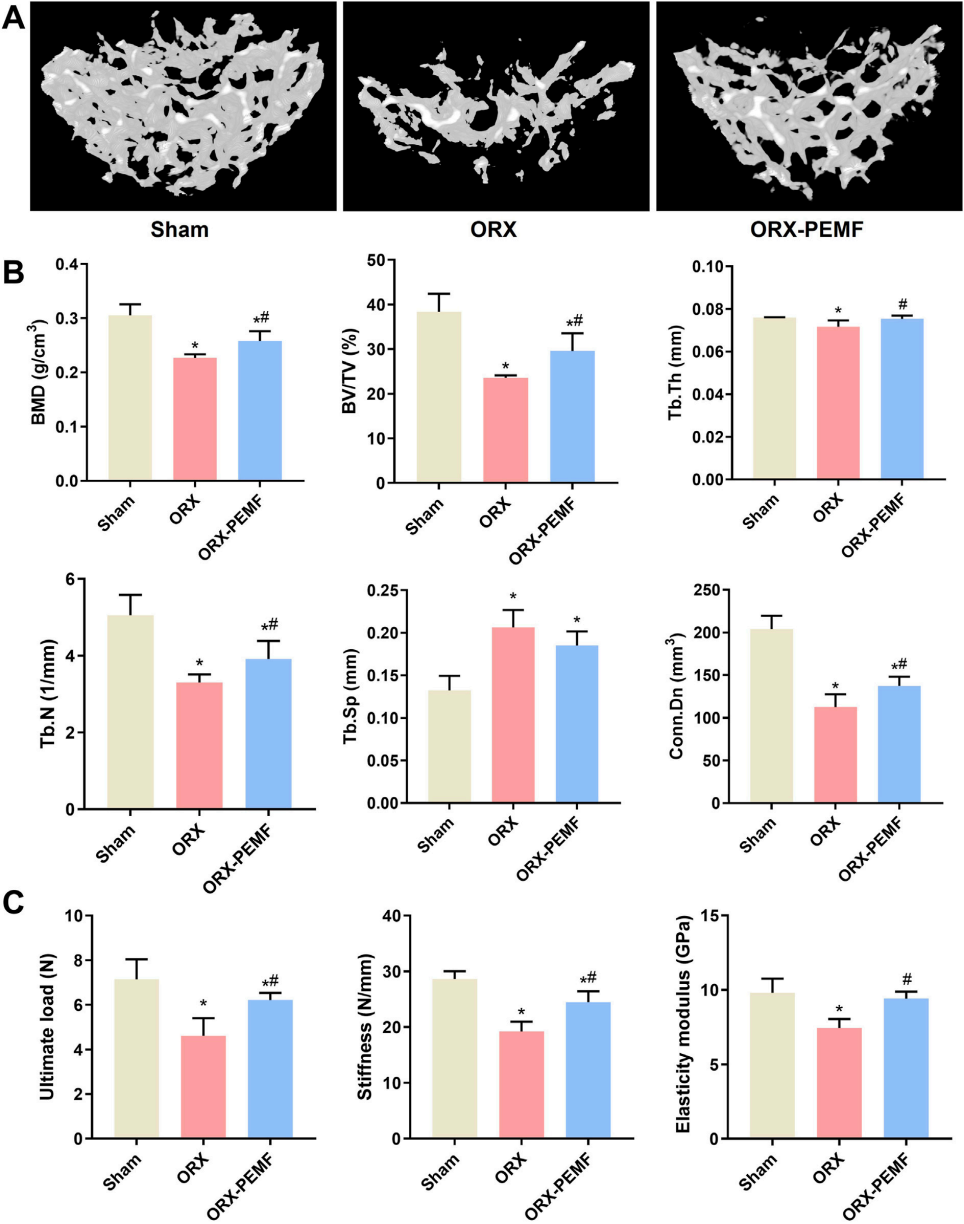
Effects of PEMF application on bone microstructure and biomechanics in the lumbar vertebra. **(A)** Three-dimensional images of trabecular bone. **(B)** Bone mineral density (BMD) and structural parameters of trabecular bone, including bone volume fraction (BV/TV), trabecular thickness (Tb.Th), trabecular number (Tb.N), connectivity density (Conn.D), and trabecular separation (Tb.Sp). **(C)** Biomechanical properties of the lumbar vertebra were evaluated, including ultimate load, stiffness, and elastic modulus. The data are shown as mean ± SD (n = 8). *p < 0.05 vs. sham, ^#^ *p* < 0.05 vs. ORX.

The micro-CT analysis of the trabecular bone in the L3 lumbar vertebra showed that the ORX procedure significantly decreased the BMD of vertebral cancellous bone and impaired the trabecular microstructure, as reflected by the significant decreases in BV/TV, Tb.Th, Tb.N, and Conn.Dn along with a significant increase in Tb.Sp compared to the sham group (Figures 2A,B). Compared to the unexposed ORX mice, the ORX mice treated with PEMF showed significant increases in L3 vertebral BMD, BV/TV, Tb.Th, Tb.N, and Conn.Dn. However, the vertebral BMD, BV/TV, Tb.N, and Conn.Dn in the ORX + PEMF group remained significantly lower than those in the sham group, while Tb.Sp remained significantly higher. These data indicate that PEMF intervention partially prevented the decrease in lumbar vertebral BMD and deterioration of the trabecular microstructure caused by ORX. Consistently, PEMF exposure significantly prevented the decreases in the ultimate load, stiffness, and elastic modulus of the L4 lumbar vertebra caused by ORX (Figure 2C).

### 3.3 PEMF application partially prevented ORX-induced reduction in bone formation in mice

**FIGURE 3:**
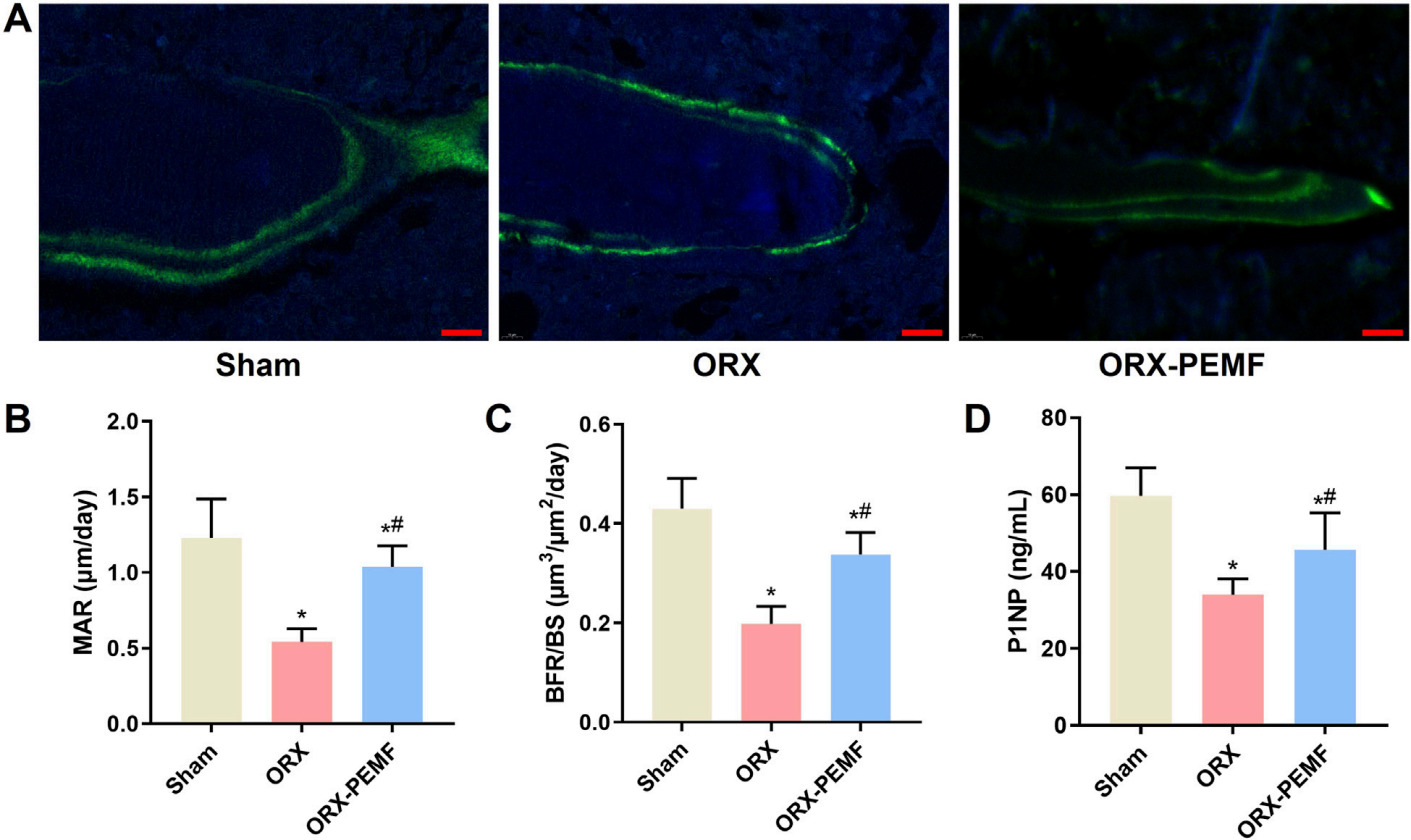
Effects of PEMF application on bone formation in ORX mice. **(A)** Representative images of bone formation marked by double-calcein labeling in the trabecular bone of the lumbar vertebra. Scale bar = 20 μm. Calculated values of the **(B)** mineral apposition rate (MAR), **(C)** bone formation rate (BFR/BS), and **(D)** concentration of serum type I procollagen N-terminal peptide (P1NP). The data are shown as mean ± SD (n = 8). *p < 0.05 vs. sham, ^#^ *p* < 0.05 vs. ORX.

The dynamic histomorphometry analysis revealed that ORX significantly reduced the MAR and BFR/BS in the L3 lumbar vertebra, whereas PEMF intervention prevented these reductions (Figures 3A–C). Additionally, PEMF application prevented the ORX-induced decrease in serum P1NP level (Figure 3D). However, compared to the sham group, the ORX + PEMF group still exhibited significantly lower MAR, BFR/BS, and serum P1NP levels. These findings indicate that PEMF treatment could partially prevent the inhibition of bone formation caused by ORX.

### 3.4 PEMF application partially prevented ORX-induced increase in bone resorption in mice

**FIGURE 4:**
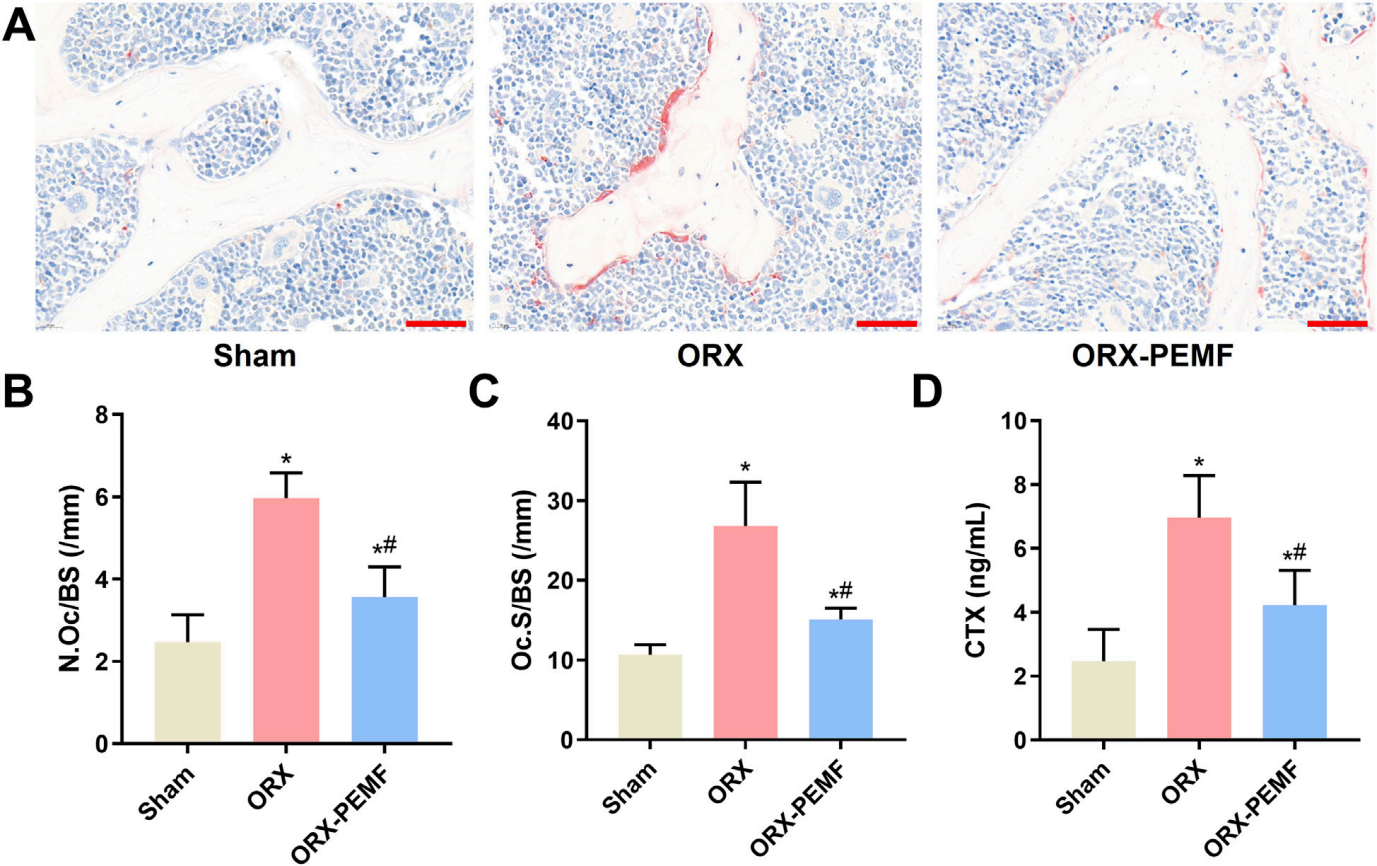
Effects of PEMF application on bone resorption in ORX mice. **(A)** Lumbar vertebra samples were TRAP stained to visualize the osteoclasts. Scale bar = 50 μm. Calculated values of the **(B)** number of osteoclasts per bone surface (N.Oc/BS); **(C)** osteoclast surface per bone surface (Oc.S/BS); and **(D)** concentration of serum C-terminal telopeptide of type I collagen (CTX). The data are shown as mean ± SD (n = 8). *p < 0.05 vs. sham, ^#^ *p* < 0.05 vs. ORX.

TRAP staining of the bone sections from the lumbar vertebrae showed that the ORX procedure significantly increased osteoclastogenesis (Figure 4A), as evidenced by the increases in N.Oc/BS and Oc.S/BS (Figures 4B,C). Compared to the unexposed ORX group, PEMF exposure significantly reduced osteoclastogenesis on the bone surface. Furthermore, PEMF treatment prevented the ORX-induced increase in serum CTX level (Figure 4D). Nevertheless, after PEMF intervention, the N.Oc/BS and serum CTX level in ORX mice remained significantly lower than those in sham mice, indicating that PEMF exposure could only partially suppress the increase in bone resorption induced by ORX.

### 3.5 PEMF application did not influence organ iron deposition in mice

**FIGURE 5:**
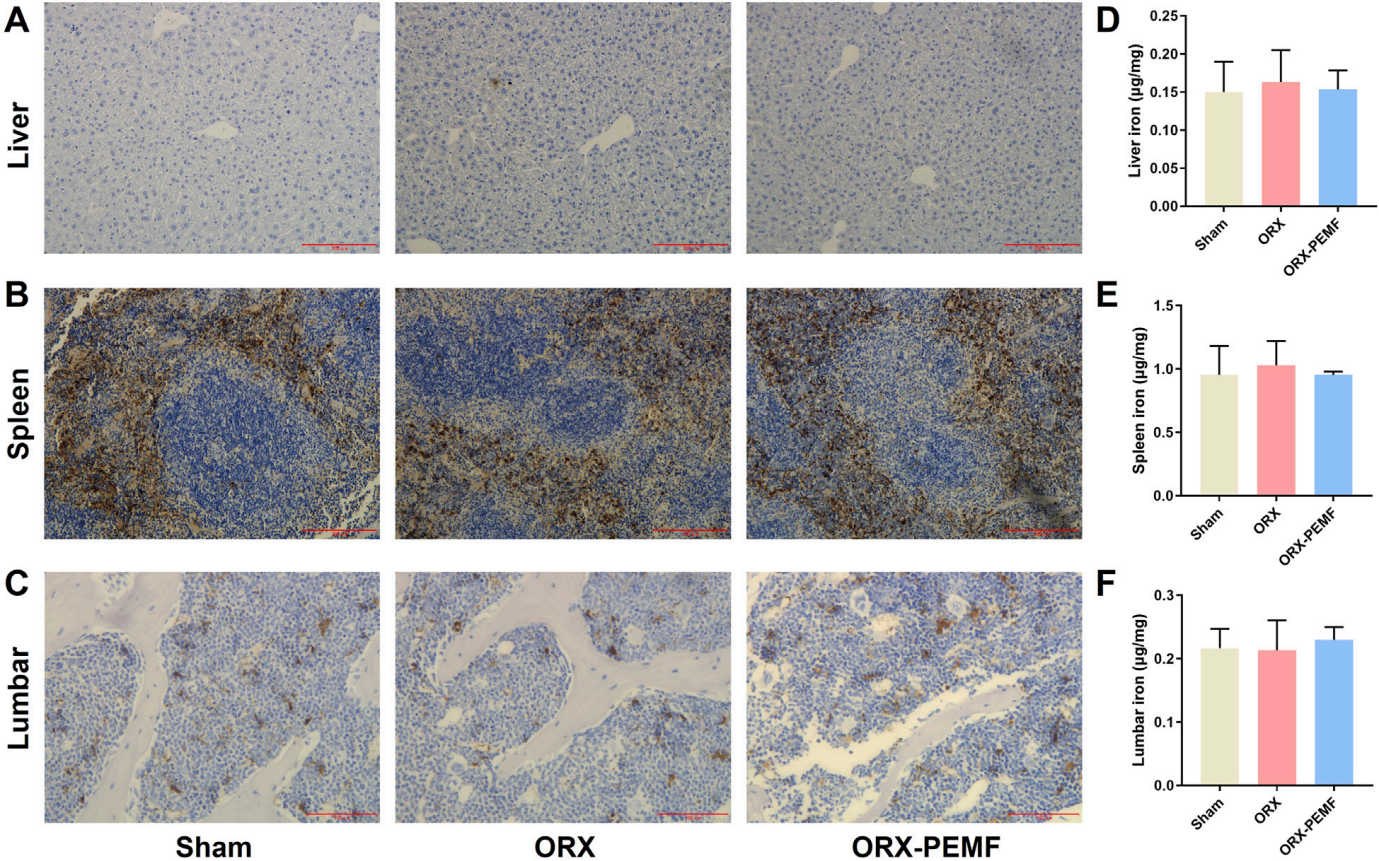
Effects of PEMF application on iron deposition in ORX mice. **(A–C)** Representative images of DAB-enhanced Prussian blue staining of the liver (scale bar: 200 μm), spleen (scale bar: 200 μm), and lumbar vertebra (scale bar: 100 μm), respectively. **(D–F)** Iron contents of the liver, spleen, and lumbar vertebra, respectively. The data are shown as mean ± SD (n = 8).

DAB-enhanced Prussian blue staining revealed that the ORX procedure did not cause significant iron deposition in the liver (Figure 5A), spleen (Figure 5B), or vertebral bone marrow (Figure 5C). Consistently, AAS analyses showed no significant changes in the iron contents in the liver, spleen, and lumbar vertebrae of ORX mice compared to sham mice (Figures 5D–F). Thus, PEMF exposure had no significant effects on iron deposition or iron contents in the liver, spleen, and lumbar bones of the ORX mice compared to untreated ORX mice, indicating that PEMF treatment did not affect organ iron deposition in the ORX mice.

### 3.6 PEMF application did not affect systemic iron metabolism in ORX mice

**FIGURE 6:**
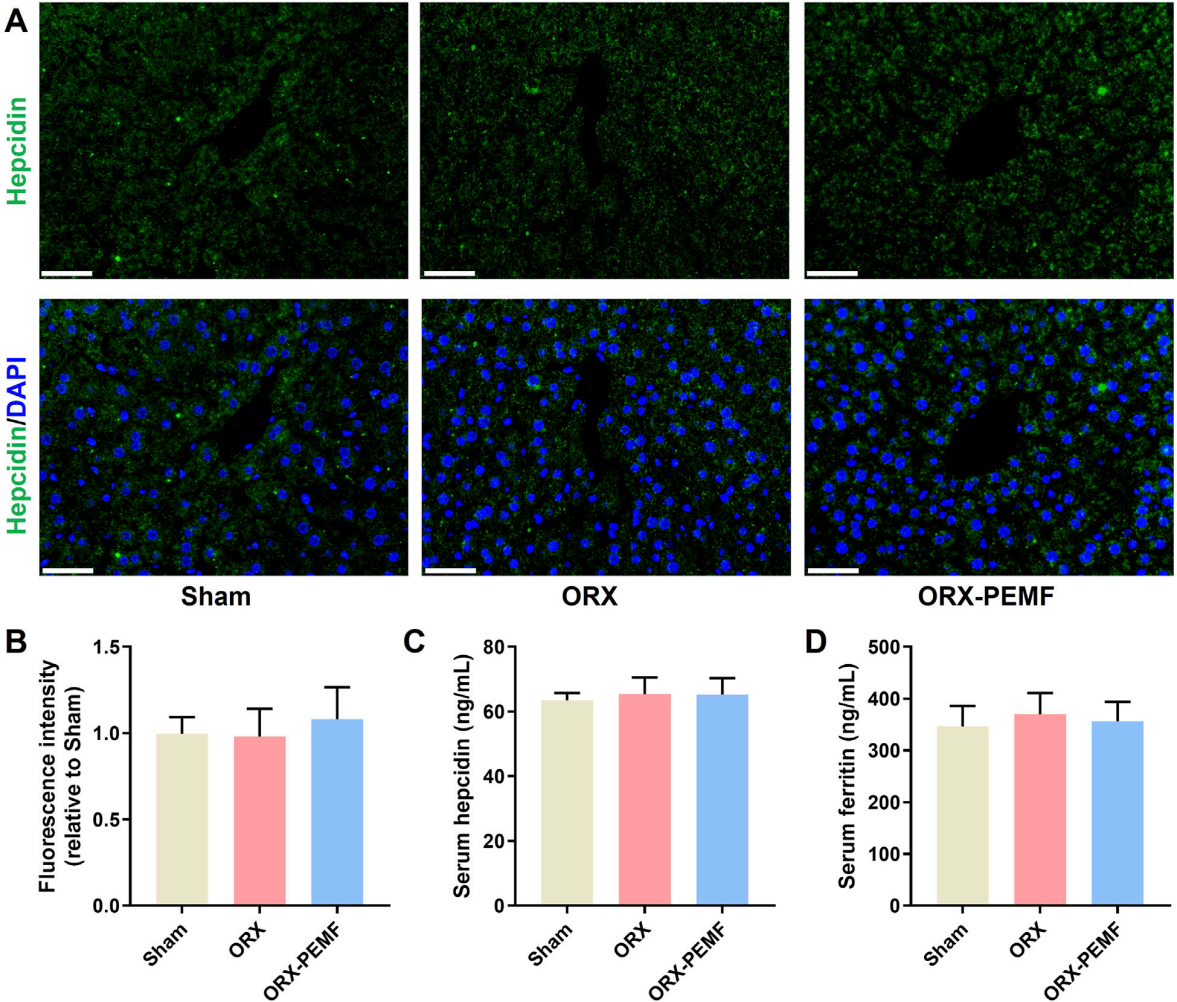
Effects of PEMF application on iron metabolism in ORX mice. **(A)** Representative immunohistochemical stained images of hepcidin in the liver. Scale bar = 50 μm. **(B)** Quantitative analyses of hepcidin fluorescence intensities relative to the sham group. **(C,D)** Serum levels of hepcidin and ferritin determined by ELISA kits, respectively. The data are shown as mean ± SD (n = 8).

Immunofluorescence staining of the liver tissue for hepcidin showed that the ORX procedure did not affect hepatic hepcidin expression and that PEMF exposure did not cause significant changes in the liver hepcidin expressions in ORX mice (Figures 6A,B). The ORX procedure did not cause significant changes in the serum levels of hepcidin or ferritin in the mice (Figures 6C,D), and PEMF exposure also had no significant effects on these serum markers. These results demonstrate that PEMF application did not impact systemic iron metabolism in the ORX mice.

## 4 Discussion

In this study, we investigated the therapeutic efficacy of PEMF application on male osteoporosis using ORX mice. The results demonstrate that PEMF exerts a protective effect on the trabecular bone in the lumbar spine. However, it shows no significant improvement against bone loss in the femur. Importantly, these effects appear to be independent of the regulation of systemic iron metabolism. This finding not only reveals the site-specific therapeutic potential of PEMF application in male osteoporosis but also provides a crucial foundation for further refinement of magnetic field treatment strategies.

Numerous *in vitro* studies have demonstrated that PEMF therapy promotes osteoblast differentiation and mineralization (Zhang et al., 2020; Miyamoto et al., 2019; Selvamurugan et al., 2017). Conversely, osteoclastogenesis and bone resorption are significantly inhibited by PEMF application (Chang et al., 2004; Chang et al., 2005; Chang et al., 2003). Therefore, the protective effects of PEMF application on lumbar vertebral trabecular bone are likely attributable to the bidirectional regulation of bone formation and resorption. Indeed, our animal experiments confirm that PEMFs partially restore MAR, BFR/BS, and serum P1NP levels in ORX mice, while concurrently suppressing osteoclast activity and attenuating the increase in serum CTX level. This suggests that PEMFs may promote a more favorable balance of bone remodeling locally within the lumbar spine by enhancing osteoblast function and inhibiting osteoclast differentiation.

Our study shows that PEMF treatment does not significantly impact femoral bone loss, suggesting that different skeletal sites may exhibit varying responsiveness to magnetic fields. As a primary weight-bearing bone, the femur undergoes bone metabolism and remodeling processes that are strongly influenced by mechanical loading, such as muscle contractions and ground reaction forces. These intense background mechanical signals may have “masked” or “interfered with” the relatively weak physical signals provided by PEMF exposure, thereby preventing the effects from becoming apparent. In contrast, the lumbar spine is subject to distinct patterns of axial mechanical loading and contains a high proportion of trabecular bone, which is highly sensitive to metabolic changes. In the absence of strong competing mechanical signals, the pro-osteogenic and antiresorptive effects of PEMFs may be more readily detectable. Therefore, future studies could investigate the effects of PEMF exposure on the femur using unloading models (e.g., hind limb unloading in mice) to isolate the influence of the mechanical load. Additionally, *in vitro* cell-based experiments may be conducted to examine whether the responses of osteoblasts and osteoclasts to PEMF exposure differ based on their tissue origins (vertebral vs. long bone).

Iron accumulation or overload is a recognized risk factor for primary osteoporosis (Che et al., 2020). The serum ferritin level serves as the gold standard indicator of iron storage in the body (Lipschitz et al., 1974). Substantial clinical data have revealed the significant inverse correlation between serum ferritin level and BMD in postmenopausal women (Lu et al., 2020; Kim et al., 2012). However, very few studies have explored the relationship between serum ferritin level and osteoporosis in men. To the best of our knowledge, the present study is a pioneering effort to investigate the impacts of PEMF application on systemic iron metabolism in male mice. The results indicate that systemic iron metabolism markers (serum hepcidin and ferritin) do not change significantly in ORX mice following PEMF treatment. It is noteworthy that some studies suggest exogenous testosterone supplementation for suppressing hepcidin expression (Bachman et al., 2010; Guo et al., 2013). This implies that the physiological regulation of hepcidin by endogenous testosterone may be pathway-dependent or subject to complex modulation by other compensatory mechanisms.

Numerous studies propose that iron metabolism may underlie the biological effects of magnetic fields (Zhen et al., 2024; Wang et al., 2025). Specifically, our previous studies showed that static magnetic fields (SMFs) can influence the biological functions of bone cells by modulating cellular iron metabolism or affect bone metabolism by regulating systemic iron metabolism (Yang et al., 2018; Dong et al., 2019; Yang et al., 2021a; Zhen et al., 2025; Zhang et al., 2023). With regard to PEMF, however, only two studies have reported modulation of iron metabolism in the neural tissues of rainbow trout and the Caribbean spiny lobster (Fitak et al., 2017; Ernst et al., 2020). The present study is therefore another pilot effort at exploring the effects of PEMFs on systemic iron metabolism in mammals. In contrast to SMFs, we observed that PEMFs did not affect hepatic hepcidin expression or lead to alterations in the circulating hepcidin and levels. A plausible explanation for this is that the magnetic flux density of an SMF is generally much higher than that typically employed in PEMF applications. Further, the PEMF frequency is another critical factor influencing its biological effects. For instance, one study showed that different PEMF frequencies exert distinct effects on osteoporotic mice (Wang et al., 2021b). Therefore, the impacts of PEMF parameter variability on systemic iron metabolism warrant further investigations.

The main limitation of the present study is the lack of exploration into the potential efficacy differences arising from different PEMF parameters (e.g., frequency, intensity, and exposure duration). Future research efforts could integrate transcriptomics or proteomics to uncover the molecular targets involved in PEMF-mediated regulation of bone metabolism as well as investigate the potential synergistic effects between PEMF and other anti-osteoporosis therapies (such as bisphosphonates or denosumab). Furthermore, given that male osteoporosis is predominantly age-related in the clinical setting, utilizing an aged osteoporotic animal model would more closely mirror the real-world application scenario. Lastly, as quadrupedal animals, mice exhibit a fundamentally distinct biomechanical loading environment in the spine compared to bipedal humans. Therefore, caution should be exercised when extrapolating these findings to the therapeutic efficacy of PEMFs in humans, which warrants further clinical validation.

In conclusion, this study confirms that PEMF application possesses therapeutic efficacy for male osteoporosis. However, this efficacy demonstrates site-specificity and appears to be independent of systemic iron metabolism regulation. These findings provide an experimental basis for the physical treatment of male osteoporosis using PEMFs, while also highlighting the need to optimize magnetic field treatment strategies based on the specific skeletal site involved and underlying pathological features.

## Data Availability

The original contributions presented in the study are included in the article/supplementary material, and any further inquiries may be directed to the corresponding authors.
